# A longitudinal surgical systems strengthening research program for medical students: the exploration of a model for global health education

**DOI:** 10.1186/s41256-021-00214-2

**Published:** 2021-09-23

**Authors:** Gregory L. Peck, Joseph S. Hanna, Erin M. Scott, Dhaval Mehta, Zina Model, Deesha Sarma, Elizabeth E. Ginalis, Zachary Berlant, Fernando Ferrera, Javier Escobar, Carlos A. Ordoñez, Carlos Morales, Vicente H. Gracias

**Affiliations:** 1 Department of Surgery, Rutgers Robert Wood Johnson Medical School, 125 Paterson Street – Suite 6300, New Brunswick, NJ 08901 USA; 2 Rutgers School of Public Health, Piscataway, NJ USA; 3 Department of Surgery, University of Massachusetts Medical School, Worcester, MA USA; 4 Department of Emergency Medicine, New York Presbyterian – Brooklyn Methodist Hospital, New York, NY USA; 5 Department of Orthopaedic Surgery, Harvard Medical School, Boston, MA USA; 6 Department of Emergency Medicine, Beth Israel Deaconess Medical Center, Boston, MA USA; 7 Department of Neurological Surgery, Rutgers Biomedical and Health Sciences, New Brunswick, NJ USA; 8 Department of Pediatrics, New York Presbyterian – Columbia University Medical Center, New York, NY USA; 9 Department of Orthopaedic Surgery, UPMC Hamot Medical Center, Pittsburgh, PA USA; 10 Rutgers Biomedical and Health Sciences, Rutgers University, New Brunswick, NJ USA; 11 Department of Surgery, Universidad del Valle, Cali, Colombia; 12 Department of Surgery, Universidad de Antioquia, Medellín, Colombia

**Keywords:** Global health, Health systems, Systems strengthening, Surgical education, Medical student education, Global surgery

## Abstract

**Background:**

In response to the staggering global burden of conditions requiring emergency and essential surgery, the development of international surgical system strengthening (SSS) is fundamental to achieving universal, timely, quality, and affordable surgical care. Opportunity exists in identifying optimal collaborative processes that both promote global surgery research and SSS, and include medical students. This study explores an education model to engage students in academic global surgery and SSS via institutional support for longitudinal research.

**Objectives:**

We set out to design a program to align global health education and longitudinal health systems research by creating an education model to engage medical students in academic global surgery and SSS.

**Program design and implementation:**

In 2015, medical schools in the United States and Colombia initiated a collaborative partnership for academic global surgery research and SSS. This included development of two longitudinal academic tracks in global health medical education and academic global surgery, which we differentiated by level of institutional resourcing. Herein is a retrospective evaluation of the first two years of this program by using commonly recognized academic output metrics.

**Main achievements:**

In the first two years of the program, there were 76 total applicants to the two longitudinal tracks. Six of the 16 (37.5%) accepted students selected global surgery faculty as mentors (Acute Care Surgery faculty participating in SSS with Colombia). These global surgery students subsequently spent 24 total working weeks abroad over the two-year period participating in culminating research experiences in SSS. As a quantitative measure of the program’s success, the students collectively produced a total of twenty scholarly pieces in the form of accepted posters, abstracts, podium presentations, and manuscripts in partnership with Colombian research mentors.

**Policy implications:**

The establishment of scholarly global health education and research tracks has afforded our medical students an active role in international SSS through participation in academic global surgery research. We propose that these complementary programs can serve as a model for disseminated education and training of the future global systems-aware surgeon workforce with bidirectional growth in south and north regions with traditionally under-resourced SSS training programs.

## Background

Global non-communicable disease care and research remains significantly under-resourced and under-studied when compared to programs concentrated on communicable disease [1]. The historical approach of developing disease-specific interventions (i.e., vertical programs) may deliver a single or limited set of interventions with selected inputs for support, but potentially creates silos which limit health system-wide development and strengthening. To address these limitations, it has been proposed that “health system strengthening” action should include the following: (1) multi-dimensional benefits which extend beyond a single disease; (2) a mechanism to address policy and organization structural constraints limiting interactions between the health system building blocks; (3) resulting in permanent systemic impact beyond the term of the project; and (4) addressing country-specific constraints and opportunities with clearly defined roles for indigenous institutions [2].

Academic collaboration through participatory action between high income and low-to-middle income countries (HICs and LMICs, respectively), and LMICs with neighbor LMICs, is necessary for the development of an objective, data-driven science which addresses these concerns. Furthermore, it is urgently needed as the global burden of conditions requiring emergency and essential surgery is rapidly becoming the world’s foremost healthcare disparity [1, 3]. Surgical system strengthening (SSS), then, is fundamental to developing sustainable emergency and essential care. However, optimal processes which promote SSS remain relatively undefined in the literature.

Nearly one-third of all United States (U.S.) graduating medical students participate in a global health elective during the course of medical school, reflecting a significant demand for global health education opportunities [4]. The parallel growth in learner interest in addressing surgical disparities, and renaissance in international advocacy and action provides a unique opportunity for learners to achieve personal development and satisfaction through participation in sustainable global health research [5]. Of 116 U.S. allopathic medical schools surveyed in one study, the majority had active student global health interest groups, of which half offered substantive transnational opportunities (including in-country research and/or clinical experiences), and over one-third offered didactic coursework in global health [6]. Despite this apparent wealth of opportunities, in a recent survey of graduating U.S. medical students, nearly half of respondents believed that global health and global surgery education in general was inadequately addressed during their training [5, 7]. This may be in part due to insufficient programmatic funding or design, inadequate development of health systems education curricula [8], or insufficient mentorship. Faculty availability to mentor learners may be limited by experience and time constraints related to professional schedules and clinical obligations for faculty and students alike. Thus, education resource limitations may be at the core of the paucity of learning opportunities cited by students.

## Objectives

Academic collaboration through participatory action research is essential to the development of surgical system science necessary for data-driven health system strengthening and promotion of sustainable global public health activities [9]. Despite this central role, recent work suggests that research opportunities may be difficult for the learners to identify and participate in [5]. Opportunities are further limited with regard to international surgical systems education through traditional undergraduate medical education (UME) pathways. To address these barriers to productive learner engagement and development within our own institution, alignment of UME global health education and longitudinal health systems research programs was established in SSS. In our institution, this was a faculty led response to the newly established Chancellor’s Rutgers Biomedical and Health Sciences Key Strategic Plan in partnership with the Rutgers Robert Wood Johnson Medical School (RWJMS), and the Division of Acute Care Surgery (ACS). In the present report, we describe this novel dedicated SSS education pathway and outcomes of this university-wide longitudinal alignment in partnership with academic institutions in Colombia, South America.

### Program design and implementation

In 2015, guided by a call to action from the Rutgers Biomedical and Health Sciences Chancellor, the Rutgers RWJMS (New Jersey, U.S.) Office of Global Health created a structured global health program to foster medical students’ active participation in global programs and allow them to graduate with honors. The program had two tracks [10]: the Distinction in Global Health (DGH) and the Chancellor’s Global Scholars (CGS). Both tracks were designed to foster formal transnational medical student education and provide an introduction to research experiences. Concomitantly, RWJMS, Universidad del Valle Medical School (HUV) in Cali, Colombia, and Universidad de Antioquia (UA) in Medellín, Colombia, executed Memoranda of Understanding outlining partnered academic commitments. The Division of ACS (RWJMS) was specifically identified as a partner for the delivery of the surgical system science research, education and mentorship aspect for these two programs.

The DGH track was designed to enroll first-year medical students who demonstrated exceptional interest in global health initiatives at the start of their medical school education. Selected students were not required to have prior experience in global health, but rather were expected to submit a feasible plan for the implementation of a global health project with the ultimate goal of directly impacting a resource-limited, at-risk, disparate, or vulnerable (domestic or international) population. While supported and directed by the Office of Global Health and an individually chosen faculty mentor, no institutional support funds were designated for DGH students. Those that fulfilled all specified requirements of the program [(1) program specific deadlines; (2) participation in one global health practicum-type experience, local or transnational; (3) presentation of a poster detailing the research experience; and (4) implementation project presented at the RWJMS University Global Health Fair] graduated with a “Distinction in Global Health” diploma notation.

The CGS track is a more intensive pathway that builds on the foundation set by the DGH requirements, and selects highly motivated students earlier in their education, and provides them with funding support. The CGS track therefore was designed to select candidate students from the accepted medical school applicant pool prior to matriculation. Eligibility criteria for the CGS program included a substantial commitment to global health, prior to medical school, demonstrated by experiential or coursework preparation. Foreign language proficiency was also expected. The students selected would be: (1) supported in their conceptualization and implementation of longitudinal global health field research projects; (2) engaged in a broad global health curriculum requiring additional didactics from the Rutgers Masters of Biomedical Sciences program in the School of Graduate Biomedical Sciences; (3) funded by the University Chancellor’s Office to coordinate two transnational practicum-type experiences, during their first and graduating year of medical school, to facilitate their research projects; and 4) expected to present their project to the global health steering committee or at a local Distinction Symposium and also graduate with the title of “Distinction in Global Health.” Aspects of each of the two tracks are compared in Table 1.

**Table 1:** Comparison of programmatic structure and characteristics for the Distinction in Global Health (DGH) and Chancellor’s Global Scholars (CGS) tracks

Program characteristic	Track	
	DGH	CGS
Eligible students	First-year medical students	Accepted medical students, prior to matriculation
Prior global health experience	Not required	Required
Guaranteed institutional funding support	No	Yes—$5000 USD
Practicum-type experience(s) required	1 experience—local or transnational	2 experiences—both transnational
Presentation of global health project at Global Health Fair	Required	Required
“Distinction in Global Health” notation on diploma upon graduation	Yes	Yes

Students with global surgery interest selected for the DGH or CGS programs were partnered with Colombian students and faculty mentors based on their specialty and regional choices. The students were mentored by international ACS faculty pairs composed of RWJMS assistant professors of surgery and Colombian university surgeons engaged in global SSS activities. Research project development and relationship building was initiated through a student performed needs assessment of a particular aspect of the Colombian surgical system. The resulting research project informed the timing and activities for the two planned international rotations (during the first and graduating years of medical school). These two one-month rotations were structured to fulfill DGH and CGS programmatic cultural immersion and research requirements [10].

The RWJMS Office of Global Health managed the four-year $5,000 USD scholarship for the CGS students. This was derived from a 40:60 split, with funding from the Chancellor’s Office, and the United States Agency for International Development—Research and Innovation Fellowship Program (USAID—RIFP; AID-OAA-A-14-00071), respectively. While there were no earmarked funds for the DGH program, accepted students were guided by their mentors in applying for grants from external and intramural sources, and were ultimately awarded a total of $1,000 USD through the Rutgers Intelligence Community Center for Academic Excellence Scholarship. The remainder of their travel costs were self-financed. The USAID—RIFP, Rutgers Global, and RWJMS Office of Global Health addressed practical logistics and student safety considerations for the rotation including, but not limited to: budget allocation, local transportation, travel and health insurance, foreign language advanced proficiency tutoring, and culturally integrative residences (e.g., housing arrangements with host families who maintain commitments to the respective local university).

Five years after inception of the DGH and CGS programs, a retrospective quantitative and qualitative review was performed to assess medical students’ level of engagement in SSS research. The scholarly productivity of the participating students was assessed as a measure of the impact of the two programs and a component of a larger evaluative process of SSS programs university wide. Ethics approval was not required for this study. Quantitatively, productivity was defined by the number of submitted regional, national and international abstracts, poster presentations, oral presentations, and submitted/published manuscripts. Scholarly work produced related to SSS research conducted in conjunction with the DGH and CGS programs was a requirement for inclusion in the analysis.

### Main achievements

In the first year of the program, three of ten (30%) DGH applicants and five of 35 (14.3%) CGS applicants (from an entering medical school class of 190) were accepted to the two programs. Three of the eight (37.5%) total accepted students in the *2015 cohort* selected a concentration in global surgery. In the second year of the program, three of seven (42.9%) DGH applicants and five of 24 (20.8%) CGS applicants (from an entering medical school class of 160) were accepted to the two programs. Again, three of the eight (37.5%) total accepted students in the *2016 cohort* selected a concentration in global surgery. In total over the two years, six global health students (one DGH and five CGS) selected a concentration in global surgery and were assigned mentors who were acute care surgeons engaged in global SSS. For a summary of application and acceptance rates, see Table 2. The remaining global health students were paired with mentors in other specialties based on their individual interests, including Obstetrics and Gynecology, Psychiatry, and Family Medicine.

**Table 2:** Applicants, acceptances and self-selected global surgery students to the Distinction in Global Health (DGH) and Chancellor’s Global Scholars (CGS) programs

Year	2015		2016	
Entering class size	190		160	
Program	DGH	CGS	DGH	CGS
Number of applicants	10	35	7	24
Number accepted	3	5	3	5
Acceptance rate	30.0%	14.3%	42.9%	20.8%
Number of accepted who self-selected global surgery mentors	1	2	0	3

The 2015 cohort students initially focused on developing institutional level processes for pre-hospital transport data collection and hospital system data collection to inform the six core surgical indicators proposed by the Lancet Commission on Global Surgery [1] to assess and inform SSS. At the end of the first year, the DGH and CGS global surgery students (in collaboration with Colombian faculty mentors) submitted the drafted needs assessment proposal to Rutgers Global and the Associate Dean of Global Health for progress evaluation and budgetary review in accordance with the awarded USAID—RIFP funding as described above. Upon approval, the three 2015 cohort students spent four weeks each translating the research conceptualization with intersectoral partners into a four-year longitudinal SSS research plan in Colombia. This was followed by a sub-analysis wherein the students and Colombian faculty identified projects suitable for individual student completion. The students gained fundamental knowledge of institutional and administrative logistics in the course of submitting a formal transnational research protocol to the respective ethics committees for approval (Table 3).

**Table 3:** List of projects produced by the students of the 2015 and 2016 cohorts

Cohort year	Student(s)	Project
2015	DS	A 2016 Pre-Implementation Study of Surgical Systems Research in Medellín, Colombia for the purposes of National Surgical, Obstetric, and Anesthesia Plans
	ZM, DM	A Stakeholder Analysis and Needs Assessment for Prehospital Data Collection in Cali, Colombia
	ZM, DM	Feasibility of Prehospital Data Collection At Handoff From EMS Providers for Trauma Systems Improvement in LMICs [13, 25]
2016	EG, ZB	The Latin American Indicator Research Coalition (LAIRC) Global Surgery Research Unit (GSRU) Model: A practical World Development Indicator data collection and implementation research tool in Medellín, Colombia
	EG, ZB	Assessing Lancet Commission on Global Surgery Indicators in Medellín, Colombia Using 2017 Global Surgery Research Units
	FF	Implementation of Global Surgery Research Unit (GSRU) for Lancet Commission on Global Surgery Core Surgical Indicator One Data Collection in Cali, Colombia

In the second year of the DGH and CGS programs, the three students in the 2016 cohort similarly spent four weeks each on coordinated implementation of SSS research to augment the work of the 2015 cohort. A needs assessment was again conducted by the students with local Colombian faculty to identify areas for improving implementation outcomes (i.e., feasibility, adaptability, cost, and appropriateness of implementation) [11] for the existing SSS research projects initially piloted by the 2015 cohort. The students: (1) presented and submitted a second surgical systems research protocol to the ethics committees and institutional review board committees of the Latin American and North American institutions respectively for approval. In conjunction with their Colombian student counterparts, the 2016 cohort adapted and implemented the projects first rolled out by the 2015 cohort, introducing a sustainable research framework for continued results and iterative productivity (Table 3).

Peer mentorship from the 2015 to the 2016 cohorts was fundamental to the continuity and sustainability of the individual research projects (Table 4). This transition was guided in large part by geographic needs and considerations. In the Medellín experience, peer mentorship facilitated a transition from a pre-implementation assessment for Lancet indicator data collection, to research protocol development, approval, and implementation with successful indicator data collection and measurement of data collection implementation outcomes. The Cali experience was characterized by expansion of the previous cohort’s established research protocol from a public institution to include a private hospital guided by the early feasibility study performed by the 2015 cohort. This was further developed in the new setting by the 2016 cohort to also measure implementation outcomes. Following the in-country research-intensive summer, each cohort resumed their regularly scheduled medical school studies while maintaining engagement in their respective projects through regular correspondence with the Colombian student and faculty teams. The DGH and CGS students from both cohorts fulfilled the requirement of presenting their research at the RWJMS University Global Health Fair.

**Table 4:** Student peer mentorship, continuation and refinement of projects

2015 cohort	2016 cohort	Site	Project evolution/development
DS	EG and ZB	Medellín, Colombia	1. Progress from pre-implementation assessment to research protocol development, IRB approval, protocol implementation, data collection 2. Increased Medellín medical student involvement and participation, including local academic presentations
ZM and DM	FF	Cali, Colombia	1. Expansion of research protocol implementation from public to include private institution participation 2. Increased Cali medical student involvement and participation, including local academic presentations

Finally, in addition to the qualitative measures of success achieved, the students participated in the drafting and acceptance of scholarly presentations. Collectively, over two years, the six students submitted five abstracts, presented six posters, delivered five podium presentations at national/international meetings, and contributed to the submission of four manuscripts (two of which have been published at the time of this study) [12, 13] related to their SSS research (Table 5).

**Table 5:** Composite academic productivity

	Abstracts	Presentations/podiums	Papers
*2015 cohort*			
DS	X	X^‡^	X
ZM	X	X^‡^	X
DM	X	X^‡^	X
*2016 cohort*			
EG	X	X^‡^	^†^
ZB	X	X^‡^	^†^
FF	X	X^‡^	^†^

X, Co-authorship/co-participation

^†^Manuscript(s) drafted

^‡^Consortium for Universities in Global Health, Latino Medical Student Association, Panamerican Trauma Society, Rutgers Global Health Fair

### Policy implications

By the year 2030, surgical disease and trauma related injuries are anticipated to become the most pressing global health need. This statistic is concerning in light of the present lack of adequate access to emergency and essential surgical care for five out of seven individuals globally [1]. This anticipated surge in need for surgical care will further strain the global community’s ability to achieve universal healthcare as proposed by the WHO without concerted efforts focused on surgical system assessment and strengthening. SSS requires a multi-pronged, multisectoral approach. In the present study, we described the outcomes of a longitudinal academic program designed to engage learners in the process of surgical system assessment and strengthening. Concomitant with their learning experience, these students were mentored to become active members and leaders in this much needed surgical system workforce expansion.

To address the paucity of structured global surgery UME curricula in the U.S., the DGH and CGS programs were developed at RWJMS to provide medical students enhanced access to education in global surgery, mentorship from faculty actively involved in SSS, the opportunity to build durable international relationships, and culminating research experiences in Latin America. To achieve SSS with Colombia, these students and their mentors developed and supported bi-directional international partnerships and parallel development of an international student cohort. Together, the DGH and CGS students championed critical issues requiring definition to support longitudinal research such as: (1) communication and collaboration between various public health stakeholders such as pre-hospital emergency medical services, and hospital- and community-based public health entities; (2) identifying host-country community/hospital needs through a participatory action research model such as desire for improved trauma registries and new processes for data quality improvement; (3) developing long-term relationships with in-country learners to build host-country research capacity and expand the growing international network of global health system scientists; and (4) fostering transnational student peer mentorship to advance research networks for students interested in global surgery from South and North America.

The benefits of including medical students as team extenders in healthcare, including improved patient care quality and educational experience, have recently been recognized [14]. Similarly, Gonzalo and colleagues proposed the value of novel roles for medical students in health systems research and capacity building [15]. This recognition has resulted in the need for assessment and innovation of current undergraduate and graduate medical education paradigms [16]. By formalizing longitudinal programs such as the DGH and CGS to realize the benefits of medical student participation in health systems action and research, these pathways can be defined and objectively refined in a critical manner with concerted education expertise.

Through the parallel DGH and CGS programs, students developed the foundational framework for sustainable, longitudinal relationships supporting health systems research between a tertiary care center focused on delivery of emergency and essential health care in North America, and similar institutions (public and private) in Medellín and Cali, Colombia. Multisectoral, multidisciplinary, and interprofessional participation in a needs assessment framed through participatory action research in Colombia was operationalized by HIC and LMIC students guided by local mentors. Students met with hospital leadership, emergency department physicians, trauma surgeons, ambulance agency leadership, secretaries of health, local medical students, and local research fellows and students to understand local SSS needs and frame research projects in response. In doing so, the learners became the “visionary and courageous leaders” who can stress the need for strong organizations and institutions, network with others to enhance shared learning, and collaborate with external donors and experts as needed to “create learning organizations” that Swanson and colleagues have advocated for [17].

During their time in Medellín and Cali, both 2015 and 2016 cohorts, along with Colombian learners and professional partners, successfully established consistent communication through web-based calls which were essential to monitor progress and problem-solving strategies with the New Jersey based Rutgers-RWJMS global surgery research team. Following the in-country research rotation, students resumed their regularly scheduled medical school studies while maintaining engagement through regular correspondence with the Colombian student and faculty teams. Both sites were successful in establishing long lasting relationships with their Colombian medical student counterparts. These relationships empowered the Colombian students to engage and participate in the research protocols at their respective sites, present abstracts and posters in Latin American symposia, and participate in reciprocal Colombian student research education rotations at RWJMS in 2016, 2017, and 2018.

The accomplishments of these first two cohorts of students are encouraging and suggest that continued support of resourced surgical systems education and research programs is warranted. It is important to recognize that by design, students accepted into these programs are self-selected, highly motivated individuals, and for the CGS students, already have a track record demonstrating their global public health priorities (i.e., international scholastic experiences). While this cohort’s engagement and productivity may not be reflective of those achieved by a wider, less experienced audience, the demonstrated value is in the opportunities for mentorship and research afforded for nascent development of SSS, and the participatory educators and researchers [18]. Therefore, while it is not surprising that these programs attract learners that are more likely to maintain engagement and strive for significant academic output with social impact, the value and need is further reflected in the proportion of students (nearly one in ten of each entering class) who pursued the opportunity to participate.

Opportunities for improvement were identified in programmatic design. For instance, each student from the HIC can spend a maximum of four weeks in the LMIC due to the medical school’s academic calendar. In the inaugural year, the time-limited student presence precluded completion of local research ethics committee approval for prehospital research protocols, ultimately delaying study implementation until the 2016 cohort was in-country. Therefore, long-term facilitation of program outcomes may require that HIC and LMIC institutions prioritize flexible student scheduling to augment depth of productivity and sustainability. A second limitation which may have hindered student productivity are the competing clinical and academic responsibilities of mentoring surgical faculty. While the learners drew substantial benefit from the ACS faculty’s expertise in systems assessment and development and local delivery of emergency and essential surgical care, this dual role at times may have also limited their time and availability to mentor energetic and innovative learners. Solutions to this vexing and well recognized issue have been difficult to identify [19], and ultimately would require systematic negotiated allowance for higher ratios of protected academic to clinical time (i.e., for research and mentorship responsibilities) for participating faculty. Finally, in the present era of health system and education cost containment in the HIC, SSS program creators and academic administrators are facing significant resource constraints affecting development of innovative education paradigms as evidenced by the disparate funding for DGH and CGS students which may limit student participation due to personal financial circumstances. It is our hope that as the impact and value of global collaboration to achieve local and international population health gains wider recognition, funding for programs such as this will increase. In fact, with new program funding, USAID has in recent years affirmed its commitment to sustainable health system strengthening, including expanding flagship programs that invest in infrastructure and frontline training for surgical and obstetric care, and support the development of health policy toward improving delivery of surgical care [21].

Insufficient academic exploration into how strengthening surgical service delivery may contribute to improved health systems outcomes has been highlighted by the Health Systems Strengthening Working Group of the Global Initiative for Emergency and Essential Surgical Care [8]. We propose that development of corresponding sister programs in LMICs may address this concern through engagement of local students, research faculty, and research institutes in academic exploration and explanation of SSS. This twinning is anticipated to maximize bidirectional research co-development [20]. Furthermore, to meet the call to action by the World Health Assembly 68.15 and 70.35 resolutions for implementation of SSS, it is essential that integrated health system education and research experiences such as those presented here have tangible culminating experiences that contribute to the development of SSS [21, 22]. Given the academic acute care surgeon’s intimate knowledge of surgical systems by way of trauma care and expertise in delivering timely emergency and essential surgical intervention, we propose that the acute care surgeon is uniquely qualified to answer this call. It is of note then that the faculty from the LMIC and HIC institutions in the present study were all acute care surgeons seeking to engage learners in the pursuit of SSS.

The RWJMS DGH and CGS programs focused on developing the future of SSS through early establishment of durable faculty-student mentor relationships, and student peer mentorship models, to create an educational environment that values both LMIC and HIC students’ research capacity in international settings [23]. The success of both programs is evident in the long-term transnational relationships that have been established, leadership development, and independent contributions to SSS disseminated through the student’s academic output. Though, further longitudinal evaluation is needed to assess the sustainability, adaptability and productivity of programs in both HIC and LMIC higher medical education. It is our hope that the creation of additional longitudinal global health tracks such as these will facilitate the identification of UME models that may contribute to the expansion of medical school global health education for proposed U.S. Liaison Committee on Medical Education (LCME) credit hour requirements [24].

## Data Availability

Data sharing is not applicable as no datasets were generated during the current study.
